# Parishin From Gastrodia Elata Ameliorates Aging Phenotype in Mice in a Gut Microbiota-Related Manner

**DOI:** 10.3389/fmicb.2022.877099

**Published:** 2022-04-25

**Authors:** Xinxiu Zhao, Shixian Zhou, Ren Yan, Caixia Gong, Qifeng Gui, Qin Zhang, Lan Xiang, Lufang Chen, Peixia Wang, Shumin Li, Yunmei Yang

**Affiliations:** ^1^Department of Geriatrics, The First Affiliated Hospital, School of Medicine, Zhejiang University, Hangzhou, China; ^2^State Key Laboratory for the Diagnosis and Treatment of Infectious Diseases, The First Affiliated Hospital, School of Medicine, Zhejiang University, Hangzhou, China; ^3^Key Laboratory of Diagnosis and Treatment of Aging and Physic-Chemical Injury Diseases of Zhejiang Province, The First Affiliated Hospital, School of Medicine, Zhejiang University, Hangzhou, China; ^4^College of Pharmaceutical Sciences, Zhejiang University, Hangzhou, China

**Keywords:** traditional Chinese medicine, natural products, antiaging, gut microbiota, metabolome

## Abstract

The physiological and pathological processes that accompany aging can seriously affect the quality of life of the elderly population. Therefore, delaying aging and developing antiaging products have become popular areas of inquiry. Gut microbiota plays an important role in age-related phenotypes. The present study aimed to investigate the antiaging effects and underlying mechanism of parishin, a phenolic glucoside isolated from traditional Chinese medicine Gastrodia elata. Samples from adult (12 weeks), low-dose (10 mg/kg/d) or high-dose (20 mg/kg/d) parishin-treated and untreated aged (19 months) mice were collected to determine blood indicators, gut microbiota and metabolome, and cardiopulmonary histopathological features. The results showed that parishin treatment ameliorates aging-induced cardiopulmonary fibrosis and increase in serum p16^*Ink*4*a*^, GDF15, and IL-6 levels. Furthermore, parishin treatment alleviated dysbiosis in gut microbiota, including altered microbial diversity and the aberrant abundance of opportunistic pathogenic bacteria such as *Turicibacter* and *Erysipelatoclostridium*. Gene function prediction and gut metabolome analysis results indicated that the parishin treatment-altered gut microbiota played important roles in sugar, lipid, amino acid and nucleic acid metabolism, and improved gut metabolic disorders in aged mice. In conclusion, the present study provides an experimental basis of potential applications of parishin against aging.

## Introduction

Along with the dramatic increase in the elderly population (≥65 years) worldwide, the world population is aging at an irreversible rate. Aging-related diseases, such as the neurodegenerative diseases Alzheimer’s disease (AD) and Parkinson’s disease (PD), diabetes mellitus (DM), tumors, and cardiovascular diseases, seriously affect the quality of life of elderly individuals and impose a huge economic burden (Li et al., 2021). Aging occurs in different physiological and pathological processes, such as tissue remodeling, injury, and cancer. Characteristics of senescent cells include persistent growth arrest, expression of anti-proliferative molecules (e.g., p16^*Ink*4*a*^), and activation of damage sensing signaling pathways. Oncogenic stress triggered by overexpression of certain oncogenes or loss of tumor suppressor genes (TSGs) in primary and tumor cells also induces senescence (Zhang et al., 2021). Therefore, delaying aging and developing antiaging products have become popular areas of inquiry.

The traditional Chinese medicine Gastrodia elata has a variety of pharmacological effects, such as anticonvulsant, analgesic, sedative, angina relief, anti-platelet aggregation, antithrombotic, anti-myocardial ischemia, anti-inflammatory, and immune enhancement effects. Parishin is a phenolic glucoside isolated from Gastrodia elata. It is a gastrodin citrate ester formed by esterification of three gastrodin molecules and the three terminal carboxyl groups of citric acid. Qi et al. confirmed that parishin exhibited antiaging effects by regulating Sir2/Uth1/TOR signaling to extend the lifespan of yeast (Lin et al., 2016). Some functional components from Gastrodia elata, such as polysaccharides, have been reported to exert a variety of health-promoting biological effects by modulating gut microbiota *in vivo* (Zhu et al., 2019; Huang et al., 2021; Huo et al., 2021), however, the antiaging effects of parishin in animal models are rarely studied. Since natural aging is a slow process accompanied by physiological and pathological changes, this study aimed to explore the health-promoting effects of long-term parishin intervention on naturally aged model.

The gut microbiota is known as the “second genome,” and there is growing evidence of a strong link between alterations in the gut microbiota and the aging process. In elderly individuals, changes in gut physiology and diet with age can lead to imbalances in immune, proinflammatory and anti-inflammatory factors, which can disrupt the gut microbiota (Ke et al., 2021). An imbalance in the gut microbiota and its metabolites may also trigger aging-related diseases and affect the aging state of an organism (Vaiserman et al., 2017). For example, the aged microbiota can promote intestinal inflammation in mice and enhance leakage of inflammatory bacterial components into the circulation (Fransen et al., 2017), and the decreased *Bacteroidetes* levels might be associated with immunosenescence among healthy middle-aged and elderly people (Shen et al., 2018)Therefore, therapies to regulate the gut microbiota and its products are essential for preventing and delaying aging. As a consequence, this study explored the relationship between the antiaging effects of parishin and its regulatory effects on the host’s gut microbiota, and provide a theoretical basis for the development and utilization of parishin.

## Materials and Methods

### Drug Preparation

Parishin was provided by Qi Jianhua’s research team at Zhejiang University (Zhejiang University Patent No. CN201610061288.2). It was dissolved in normal saline and prepared as a solution with a concentration of 2 mg/ml.

### Animal Experimental Design

SPF-grade C57BL/6 male adult mice (12 weeks, weight 20 ± 2 g) and aged mice (19 months, 20–30 g) were provided by Zhejiang Experimental Animal Center, and all the mice were fed a normal diet and housed at room temperature with 12/12 h day/night alternation. Due to the lack of *in vivo* studies on the antiaging effects of parishin, the dose setting of parishin in this study was based on the results of *in vitro* experiments (Lin et al., 2016) and with reference to the reported study of gastrodin in the long-term treatment of natural aging-related disease (Huang et al., 2015). After 2 weeks of acclimatization, the mice were randomly divided into four groups (*N* = 10, 5 per cage) as follows (Charan and Kantharia, 2013): the young group (YC), adult mice were gavaged with saline; the old group (AC), aged mice were gavaged with saline; the low-dose group (LPar), aged mice were gavaged with parishin (10 mg/kg/d); and the high-dose group (HPar), aged mice were gavaged with parishin (20 mg/kg/d). The treatment lasted for 8 weeks, once daily, and the feces of all the groups were collected at the end of 8 weeks. After completion of the treatment, the mice were anesthetized with pentobarbital sodium before sacrifice. Eye blood was taken for hematological testing, and heart and lung tissues were taken for histopathological evaluation. All the experimental procedures followed the National Institutes of Health (NIH) Guide for the Care and Use of Laboratory Animals. The study protocol was approved by the Animal Experimentation Ethics Committee of Zhejiang University (no. 1446).

### Blood Indicator Testing

The blood samples were allowed to stand for 30 min at room temperature, and the serum was separated by centrifugation at 3,000 rpm for 10 min. The serum levels of GDF15, p16^*Ink*4*a*^, and IL-6 were measured by ELISA (Cusabio Biotech, Wuhan, China).

### Fecal 16S rDNA Sequencing and Analysis

Fecal DNA was extracted using the QIAamp Fast DNA Stool Mini Kit (Qiagen, Valencia, United States). PCR amplification of the 16S rDNA variable region (V3 + V4) was performed using primers 338F (5′-ACTCCTACGGGAGGCAGCAG-3′) and 806R (5′-GGACTACHVGGGTWTCTAAT-3′; Lv et al., 2021b). The PCR products were purified using AMPure XPbeeds (Agencourt, Beckman Coulter, United States), and the library was quantified by real-time quantitative PCR. Paired-end sequencing (2 × 300 bp) was performed using the Illumina MiSeq platform (Illumina, San Diego, CA). FLASH (v1.2.8) software was used to clean, filter and merge the raw reads. Vsearch (v2.3.4) was then used to select operational taxonomic units (OTUs) with > 97% sequence similarity. OTU clustering and identification based on the RDP database and the NCBI-16S database as well as microbial diversity and differential enrichment were analyzed using QIIME (v1.8.0). PICRUSt2 was used to predict the gene function of the obtained OTUs.

### Fecal Metabolome Analysis

Sample preparation for metabolic assays was performed as described previously (Lv et al., 2021a). Briefly, a mixture of 20 mg feces and 800 μL methanol was homogenized three times using a Precellys Evolution instrument (Bertin Technologies, United States). After centrifugation at 14,000 rpm for 15 min, the supernatant was filtered through a 0.22 μm membrane, and 20 μL of heptadecanoic acid (1 mg/mL, Sigma–Aldrich, St. Louis, MO, United States) was added as an internal reference and then dried under nitrogen at room temperature. After drying, the samples were methoxymated with methoxypyridine (20 mg/mL, Sigma–Aldrich, St. Louis, MO, United States) and trimethylsilylated with N,O-bistrifluoroacetamide containing 1% trimethylsilyl chloride. The pretreated samples were analyzed with an Agilent 7890A-5975C GC–MS system (Agilent, United States). The downstream data were compared with NIST 17 databases to identify the corresponding metabolites (matching score ≥ 80%).

### Histopathological Examination

The mouse heart and lung tissues were fixed in 4% paraformaldehyde (Beijing Solarbio Technology Co., Ltd.) at room temperature for 24 hours and then embedded in paraffin and cut into 4-μm-thick sections. Hematoxylin and eosin (H&E) and Masson’s trichrome staining were used to visualize the architecture and fibrosis of cardiac and lung tissues, respectively. The slices were examined under an inverted light microscope (Leica, Berlin, Germany). The degree of fibrosis was quantified by ImageJ software and expressed as a percentage of the fibrotic area of the whole region.

### Statistics

For the comparison of serum GDF15, p16*^Ink4a^*, and IL-6 levels, quantitative cardiac and lung histopathology results and intestinal bacterial alpha diversity and fecal metabolites between groups, the Shapiro–Wilk method was first used to test whether the data of each group conformed to a normal distribution. Linear mixed models was used to compare any two data sets that were normally distributed; otherwise, the Mann–Whitney *U* test was used. The Wilcoxon rank sum test combined with the Benjamini–Hochberg method was used to compare the relative abundance of each taxonomic level of intestinal bacteria between groups. Correlation analysis between variables was performed using Spearman’s rank correlation test. *P* < 0.05 was considered a statistically significant difference.

## Results

### Parishin Treatment Ameliorates Aging-Induced Cardiopulmonary Injury and Changes in Serum Biomarker Expression

**FIGURE 1:**
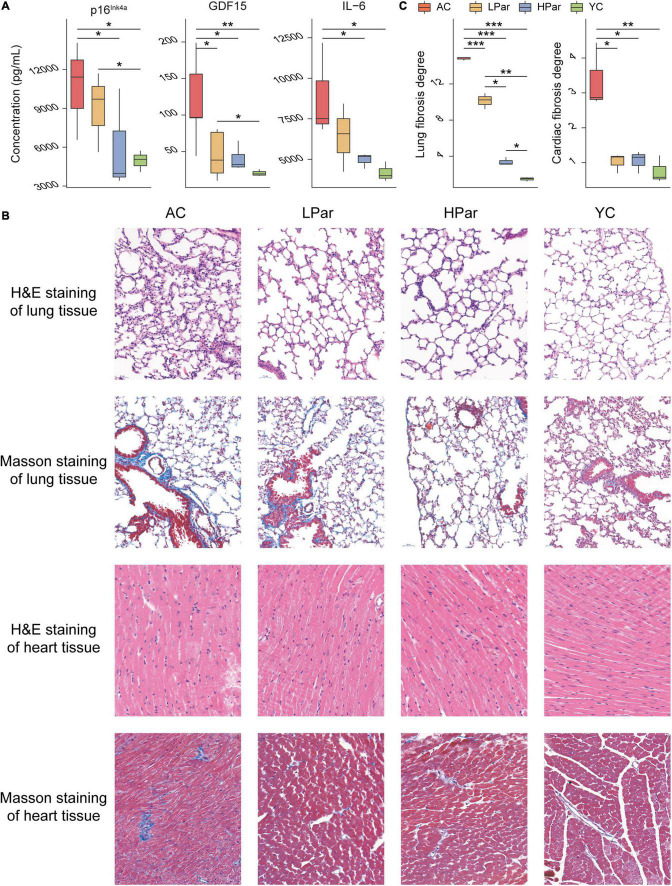
Parishin treatment alleviates aging-induced abnormal blood indicator levels and cardiopulmonary injury. **(A)** Serum p16^*Ink*4*a*^, GDF15, and IL-6 levels. **(B)** Representative images of lung and heart samples stained by H&E and Masson’s trichrome. **(C)** Fibrosis degree of lung and heart samples. *P < 0.05; **P < 0.01; ***P < 0.001.

To investigate the ameliorative effect of parishin on the aging phenotype in mice, we examined changes in some important aging-related physiopathological indicators after parishin treatment. The ELISA results showed that the serum p16^*Ink*4*a*^, GDF15, and IL-6 levels were significantly lower in the parishin-treated mice (especially in the HPar group) than in the AC group mice (Figure 1A). In the lung tissue, H&E staining showed that the alveoli were collapsed, the disturbed alveolar structure, inflammatory cells infiltration and fibroblasts proliferated were observed in the AC group mice compared with the YC group mice; In the LPar and HPar groups, the morphological structure of the lung tissue was improved, the number of infiltrating inflammatory cells was decreased, and alveolar structure was restored. Masson staining showed a large number of blue-stained collagen fibers in the AC group. In the parishin treatment groups, collagen deposition was reduced (Figure 1B). In the heart tissue, H&E staining showed that cardiomyocytes presented a disordered arrangement in the AC group compared to the YC group. The cardiomyocyte arrangement became significantly ordered in the LPar and HPar mice, especially in the HPar treatment group. Masson staining showed a large area of collagen accumulation in the AC group, which indicated a high level of fibrosis, while parishin treatment alleviated collagen accumulation in the LPar and HPar groups. Additionally, the fibrosis degree analysis confirmed that parishin treatment improved the cardiopulmonary fibrosis in aged mice (Figure 1C).

### Parishin Treatment Ameliorates Aging-Induced Changes in the Gut Microbiota

**FIGURE 2:**
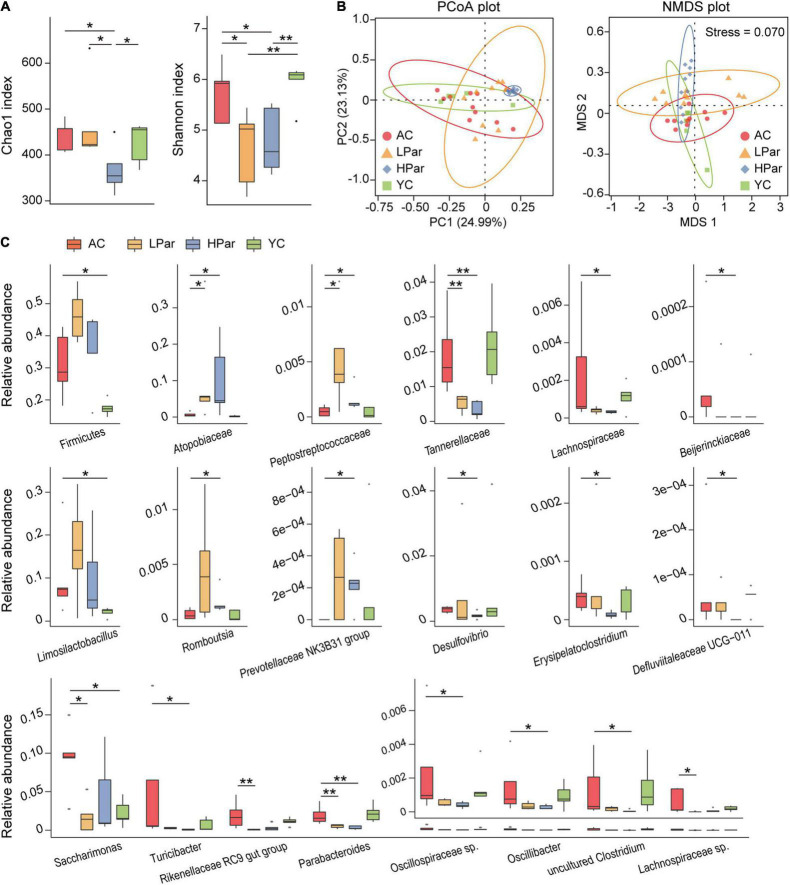
Parishin treatment alleviates aging-induced dysbiosis of the gut microbiota. **(A)** Box plot of community biodiversity and richness estimated based on the Shannon indexes and Chao1 indexes. **(B)** Two-dimensional PCoA and NMDS plot based on the Bray–Curtis dissimilarity. **(C)** Alterations in the relative abundance of bacterial taxa in the AC, LPar, HPar and YC groups. *P < 0.05; **P < 0.01.

Changes in structure and function of gut microbiota accompany the aging of gut tissue. To investigate the regulatory effect of parishin on the gut microbiota, we quantitatively determined and analyzed the gut bacteria of each group of mice based on 16S rDNA sequencing. A total of 1,304,626 reads from 40 fecal samples were obtained by 16S rDNA sequencing. Alpha diversity analysis showed that the community biodiversity indicated by the Shannon index and the community richness indicated by the Chao 1 index were not significantly different between the YC and AC groups, while the community biodiversity and community richness in the HPar and LPar groups were significantly changed compared to those in the YC and AC groups (Figure 2A). For beta diversity, microbial community dissimilarities among the four groups of mice were visualized by principal coordinate analysis (PCoA) and non-metric multidimensional scaling (NMDS) plots (Figure 2B), and the significance of intergroup differences between groups was determined by analysis of similarities (ANOSIM) and Adonis. The results showed significant differences in all comparisons except HPar vs. LPar. (Table 1).

**TABLE 1:** Analysis of similarities (ANOSIM) and Adonis analysis results based on Bray–Curtis dissimilarity (permutations = 999).

	ANOSIM		Adonis	
		
	*R*	*P* value	*R* ^2^	Pr (>F)
AC vs. YC	0.252	0.019	0.210	0.015
AC vs. LPar	0.384	0.014	0.241	0.009
AC vs. Hpar	0.448	0.008	0.239	0.009
YC vs. LPar	0.560	0.007	0.320	0.001
YC vs. HPar	0.596	0.008	0.305	0.001
HPar vs. LPar	0.048	0.241	0.129	0.246
AC vs. LPar vs. Hpar vs. YC	0.383	0.001	0.322	0.001

We further analyzed the changes in the relative abundance of individual bacterial taxa between the YC, AC, HPar and LPar groups. The phylum Firmicutes and genera *Limosilactobacillus* and *Saccharimonas* were enriched in the AC group compared with the YC group. The abundance of bacterial taxa from Firmicutes or *Limosilactobacillus* showed an increasing trend after parishin treatment compared to the AC group, while the abundance of *Saccharimonas* in the LPar group decreased significantly compared to that in the AC group (*P*_*adj*_ = 0.016). In addition, the abundance of bacterial taxa from families *Atopobiaceae* and *Peptostreptococcaceae* and genera *Prevotellaceae* NK3B31 group and *Rombousia* were enriched in the LPar and/or HPar group compared to the AC group, while the families *Tannerellaceae*, *Lachnospiraceae*, and *Beijerinckiaceae* and the genera *Erysipelatoclostridium*, *Defluviitaleaceae* UCG-011, *Turicibacter*, *Rikenellaceae* RC9 gut group, *Parabacteroides*, *Oscillospiraceae* sp., *Oscillibacter*, *Lachnospiraceae* sp., *Desulfovibrio* and an uncultured *Clostridum* were depleted (Figure 2C).

### ParIshin-Altered Gut Microbial Function Is Mainly Associated With Sugar, Lipid, Amino Acid and Nucleic Acid Metabolism

**FIGURE 3:**
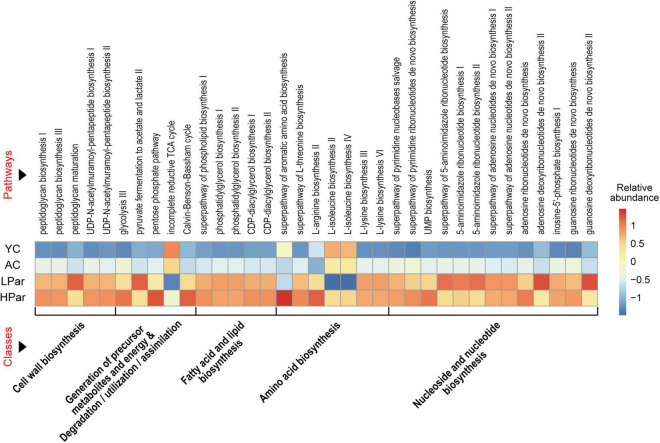
Pathway enrichment of differential gut microbial genes between the YC, AC, LPar and HPar groups based on function prediction using Picrust 2 (*P* < 0.05).

To explore the possible health-promoting role of gut microbial alterations induced by parishin teatment, we predicted the functions of the differential gut microbes between the YC, AC, LPar and HPar groups by using Picrust 2 software and then mapped the differential functional genes to the MetaCyc pathway database and clustered them according to abundance. As shown in Figure 3, these differentially expressed functional genes were mainly enriched in five classes of cell wall biosynthesis, generation of precursor metabolites and energy and degradation/utilization/assimilation, fatty acid and lipid biosynthesis, amino acid biosynthesis, and nucleoside and nucleotide biosynthesis for a total of 35 pathways (*P* < 0.05).

**FIGURE 4:**
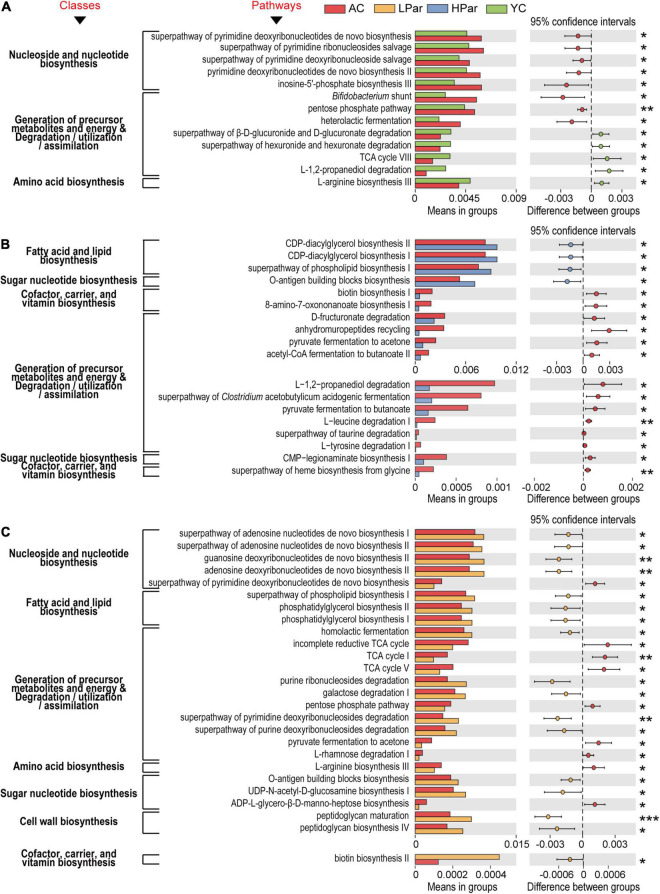
Pathways of gut microbial genes altered by aging **(A)** and whose alterations were alleviated by high-dose parshin treatment **(B)** and low-dose parshin treatment **(C)**. *P < 0.05; **P < 0.01; ***P < 0.001.

We further analyzed the functional differences in the differentially enriched gut microbes in each comparison. In the comparison of AC and YC, differential functional genes located in nucleoside and nucleotide biosynthesis-related pathways, including the superpathway of pyrimidine deoxyribonucleotide *de novo* biosynthesis, superpathway of pyrimidine ribonucleosides salvage, superpathway of pyrimidine deoxyribonucleoside salvage, pyrimidine deoxyribonucleotide *de novo* biosynthesis II and inosine-5’-phosphate biosynthesis III pathways, and genes located in the generation of precursor metabolites and energy and degradation/utilization/assimilation-related pathways, including bifidobacterial shunts, pentose phosphate pathway and heterolactic fermentation pathway, were significantly enriched; however, genes located in the superpathway of β-D-glucuronide and D-glucuronide degradation, superpathway of hexuronide and hexuronate degradation, TCA cycle VIII and L-1,2-propanediol degradation pathway, and an amino acid biosynthesis-related pathway, L-arginine biosynthesis III, were significantly reduced (Figure 4A).

After high-dose parishin treatment, all nucleoside and nucleotide biosynthesis-related pathways in which the differentially expressed functional genes that located in the AC vs. YC were no longer significant. In addition, the differentially expressed genes that were significantly downregulated in the HPar group were mainly located in fourteen cofactor, carrier and vitamin biosynthesis-, precursor metabolite and energy production and degradation/utilization/assimilation-, and sugar nucleotide biosynthesis-related pathways, such as biotin biosynthesis I, L-tyrosine degradation I, and CMP-legionaminate biosynthesis I, while the significantly upregulated genes in the HPar group were located in the O-antigen building blocks biosynthesis pathway and fatty acid and lipid biosynthesis-related pathways, such as CDP-diacylglycerol biosynthesis I and II, and superpathways of phospholipid biosynthesis I (Figure 4B).

However, the LPar group exhibited upregulated differentially expressed genes in four nucleoside and nucleotide biosynthesis-related pathways, including the superpathways of adenosine nucleotide *de novo* biosynthesis I and II, guanosine deoxyribonucleotide *de novo* biosynthesis II, and adenosine deoxyribonucleotide *de novo* biosynthesis II, compared to the AC group, while the downregulated genes were located in the superpathway of pyrimidine deoxyribonucleotide *de novo* biosynthesis (Figure 4C). On the other hand, similar to the those associated with the high-dose parishin treatment, the upregulated genes associated with the low-dose parishin treatment were also located in the O-antigen building block biosynthesis pathway and in three fatty acid and lipid biosynthesis-related pathways, namely, phosphatidylglycerol biosynthesis I and II and the superpathway of phospholipid biosynthesis I. Additionally, the altered genes associated with low-dose parishin treatment were mainly located in seventeen cell wall biosynthesis-, cofactor, carrier, and vitamin biosynthesis-, generation of precursor metabolites and energy and degradation/utilization/assimilation-, amino acid biosynthesis-, and sugar nucleotide biosynthesis-related pathways, such as peptidoglycan biosynthesis IV, biotin biosynthesis II, TCA cycle, L-arginine biosynthesis III and UDP-N-acetyl-D-glucosamine biosynthesis I (Figure 4C).

### Parishin Treatment Improves Gut Metabolic Disorder

**FIGURE 5:**
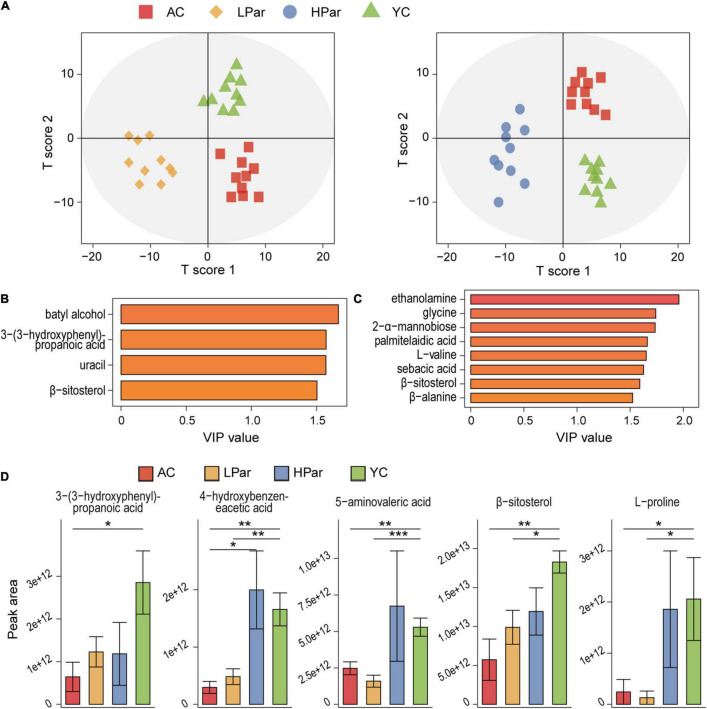
Parishin alleviates aging-induced gut metabolic disorder. **(A)** OPLS-DA plot illustrating clear separation of gut metabolic profiles of the AC, LPar and YC groups, and the AC, HPar and YC groups. **(B)** VIP values of 4 metabolites with the highest contribution to the separation of the AC, LPar and YC groups in the OPLS-DA model. **(C)** VIP values of 8 metabolites with the highest contribution to the separation of the AC, HPar and YC groups in the OPLS-DA model. **(D)** Levels of five differentially distributed metabolites in the four groups. *P < 0.05; **P < 0.01; ***P < 0.001.

Abnormal gut metabolism is one of the common aging phenotypes, and the co-metabolites between gut microbes and the host are the bridge for their interactions. To clarify the effect of parishin treatment on aging-induced gut metabolic alterations, we quantified fecal metabolites in mice using gas chromatography–mass spectrometry (GC–MS). A total of 66 metabolites were identified from 40 fecal samples. The results of orthogonal projections to latent structures discriminant analysis (OPLS-DA) showed a clear separation between the YC, AC and LPar groups and between the YC, AC and HPar groups (Figure 5A), indicating significant differences in their metabolomic profiles. When using the values of variable importance for projection (VIP) > 1.5 as a threshold, the metabolites with significant contributions to differentiate the fecal metabolome profiles of the YC, AC and LPar groups were batyl alcohol, 3-(3-hydroxyphenyl)-propanoic acid, uracil and β-sitosterol (Figure 5B); the metabolites that contributed significantly to the fecal metabolome profiles of the YC, AC and HPar groups were ethanolamine, glycine, 2-α-mannobiose palmitelaidic acid, L-valine, sebacic acid, β-sitosterol and β-alanine (Figure 5C).

Next, we compared the fecal metabolite levels between each group. The levels of 3-(3-hydroxyphenyl)-propanoic acid, L-proline, 4-hydroxybenzeneacetic acid, 5-aminovaleric acid and β-sitosterol were significantly lower in the AC group than in the YC group. After treatment with high-dose parishin, the levels of these five metabolites were restored and no longer significantly different from those in the YC group; however, only the levels of 3-(3-hydroxyphenyl)-propanoic acid were recovered after low-dose parishin treatment (Figure 5D).

### Parishin-Altered Gut Microbiota, Metabolites, and Aging-Related Indicators Are Closely Correlated

**FIGURE 6:**
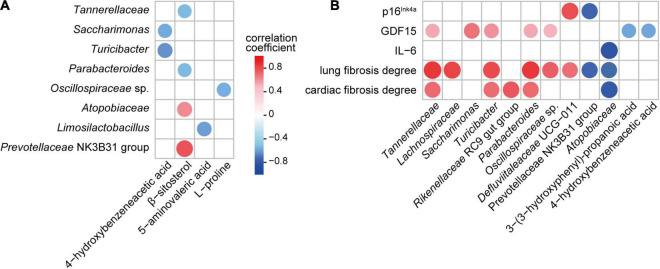
Associations among gut bacteria, metabolites, and aging-related indicators influenced by parishin (*P* < 0.05). **(A)** Correlation of parishin-influenced gut microbes with gut metabolites. **(B)** Correlation of parishin-influenced microbes and metabolites with serum biomarkers and cardiopulmonary fibrosis degrees.

To explore the potential relationship between the gut microbiota and metabolome, Spearman’s rank correlation test was used to analyze the correlations between significantly altered bacterial taxa and metabolites in the feces. Both the absolute value of the correlation coefficient *r* > 0.4 and *P* < 0.05 were used as the screening threshold. The results showed that *Turicibacter* and *Saccharimonas* were negatively associated with 4-hydroxybenzeneacetic acid; *Atopobiaceae* and *Prevotellaceae* NK3B31 group were positively associated with β-sitosterol; *Tannerellaceae* and *Parabacteroides* were negatively correlated with β-sitosterol; *Limosilactobacillus* was negatively correlated with 5-aminovaleric acid; and Oscillospiraceae sp. was negatively correlated with L-proline (Figure 6A).

Next, we analyzed the association of gut microbiota and metabolites with aging-related indicators in mice. The results showed that GDF15 was positively correlated with *Tannerellaceae*, *Saccharimonas*, *Turicibacter*, *Parabacteroides* and *Oscillospiraceae* sp. and negatively correlated with 3-(3-hydroxyphenyl)-propanoic acid and 4-hydroxybenzeneacetic acid; p16^*Ink*4*a*^ was negatively correlated with the *Prevotellaceae* NK3B31 group and positively correlated with *Defluviitaleaceae* UCG-011; IL-6 was negatively correlated with *Atopobiaceae*; lung fibrosis degree was positively correlated with *Tannerellaceae*, *Lachnospiraceae*, *Turicibacter*, *Parabacteroides*, *Oscillospiracea*e sp. and *Defluviitaleaceae* UCG-011 and negatively correlated with *Prevotellaceae* NK3B31 group and *Atopobiaceae*; and the cardiac fibrosis degree was positively correlated with *Tannerellaceae*, *Turicibacter*, *Rikenellaceae* RC9 gut group and *Parabacteroides* and negatively correlated with *Atopobiaceae* (Figure 6B).

## Discussion

Aging is one of the greatest risk factors for chronic diseases, including cardiovascular diseases, metabolic disorders, neurodegenerative diseases and various malignancies as well as respiratory diseases such as chronic obstructive pulmonary disease and interstitial pulmonary fibrosis, which together constitute the majority of global morbidity, mortality and healthcare costs (Martel et al., 2020). The problems associated with aging gradually lead to a decline in the function of almost all organisms (Campisi et al., 2019). Gastrodia elata is a commonly used traditional Chinese medicine with various pharmacological effects. The antiaging functions of bioactive ingredients in Gastrodia elata, such as gastrodin, have been widely reported in terms of life extension, neuroprotection, antiinflammation and antioxidation (Chen et al., 2021; He et al., 2021; Wang et al., 2021). However, most studies focus on the therapeutic effects of these bioactive ingredients on aging-related diseases, therefore, animal models with artificially induced pathological damage are often used. Natural aging is a slow process accompanied by physiological and pathological changes. In this study, we focused on the health-promoting effects of long-term parishin intervention during natural aging, and attempted to correlate it with the regulatory effects of parishin on other aging phenotypes such as changes in gut microbiota and metabolism to reveal its possible underlying mechanism.

Aging impacts gut integrity and can be causally linked to changes in the microbiota (DeJong et al., 2020), and prevention of aging-related changes in intestinal physiology mitigates microbial dysbiosis and extends lifespan (Li et al., 2016). Many studies have shown that the gut microbiota plays an important role in aging-related diseases and unhealthy aging. For example, gut dysbiosis contributes to Alzheimer’s disease pathologies by enhancing neuroinflammation and regulating microglia activation in the brain (Chen et al., 2022); age-related changes in the gut microbiota influence systemic inflammation and stroke outcome (Spychala et al., 2018). Transplantation of gut microbes from aged mice to adult mice induces an obese phenotype, promotes inflammatory responses, impairs spatial memory and learning ability, and causes an aging phenotype in adult mice, suggesting the possibility of adaptive changes in gut microbiota in response to the aging process and the possibility of life extension by modulating the gut microbiota (Finlay et al., 2019). A previous study suggested that parishin alleviates the aging phenotype, but the mechanism of action needs to be explored. In this study, we found that the antiaging properties of parishin may be closely related to its regulatory effects on host gut microbiota and metabolism. These results provide a preliminary basis for the use of parishin in antiaging therapy. Our results showed that parishin treatment ameliorated pathological damage to the heart and lung and elevated serum GDF15, IL-6 and p16^*Ink*4*a*^ levels in aged mice. Senescent cells produce a unique secretory phenotype, the senescence-associated secretory phenotype (SASP), which consists of various cytokines, chemokines, growth factors, proteases, lipids and non-macromolecular elements (Birch and Gil, 2020). The aging process is accompanied by the progressive development of chronic systemic inflammation (inflammatory aging). GDF15 is associated with inflammation and is thought to be a stress-inducing factor. The p16^*Ink*4*a*^ protein induces cellular aging and inhibits infinite cell division, thereby maintaining cell cycle homeostasis, and it is one of the key molecular markers of cellular senescence (Liu et al., 2019). The multifunctional cytokine IL-6 also plays an important role in the aging process, and the elevated expression of IL-6 in aged cell tissues accelerates aging and induces the development of age-related diseases. Parishin treatment significantly ameliorated aging-induced increases in GDF15, IL-6 and p16^*Ink*4*a*^ levels, suggesting a better modulation of aging-associated abnormalities in the cell cycle and inflammation levels. These results are consistent with our finding that parishin alleviates aging-induced cardiopulmonary histopathological damage.

Our results showed that parishin alleviated the decline in some potentially beneficial microbes, such as *Atopobiaceae* and *Prevotellaceae*, and the enrichment of some conditionally pathogenic bacteria, such as *Clostridum*, *Turicibacter*, and *Erysipelatoclostridium*, during senescence. *Atopobiaceae* may be associated with protection against multidrug-resistant organism colonization in elderly individuals (Ducarmon et al., 2021). The enrichment of fecal *Prevotellaceae* and the depletion of *Rikenellaceae* and *Clostridum* have been reported to play a role in the maintenance of high physical function in older adults (Fielding et al., 2019). *Turicibacter* has been reported to be positively related to the enhanced immune response (Su et al., 2018) and associated with host inflammation (Chung et al., 2021). Fecal *Erysipelatoclostridium* was enriched and increased with greater gastrointestinal dysfunction and contributed to increased production of tryptophan in patients with Parkinson’s disease (Rosario et al., 2021). Our results of the correlation analysis are consistent with these findings; for example, potentially beneficial bacteria, such as the *Atopobiaceae* and *Prevotellaceae* NK3B31 group were negatively correlated with IL-6 and p16^*Ink*4*a*^ protein expression and cardiopulmonary fibrosis degrees, while potentially harmful bacteria, such as *Turicibacter* and *Tannerellaceae*, were positively correlated with GDF15 and cardiopulmonary fibrosis degrees. These results indicate that regulation of gut microbial composition may be one of the important means by which parishin exerts its anti-aging effects.

The functional prediction results suggest that the significantly changed gut microbes after parishin treatment may play important roles in pathways including the generation of precursor metabolites and energy and degradation/utilization/assimilation, amino acid biosynthesis, and cofactor, carrier, and vitamin biosynthesis. These findings are consistent with the results of the fecal metabolome analysis in which parishin treatment reversed the aging-induced depletion of 3-(3-hydroxyphenyl)-propanoic acid, L-proline, 4-hydroxybenzeneacetic acid and 5-aminovaleric acid. 5-Aminovaleric acid, the γ-aminobutyric acid (GABA) analog, is an important intermediate in the pathways of L-lysine degradation and L-proline degradation (Burgardt et al., 2021; Cheng et al., 2021); the microbial flavonoid metabolite 4-hydroxybenzeneacetic acid, which possesses anxiolytic effects, is generated from gut microbial polyphenol and amino acid metabolism, and is an important intermediate in the pathways of L-tyrosine degradation and automatic biogenic amine degradation (Zabela et al., 2016; Orabi et al., 2021); 3-(3-hydroxyphenyl)-propanoic acid, which has been reported to exhibit hepatoprotective activities by regulating oxidative stress, lipid peroxidation, and inflammatory responses (Cui et al., 2018), is an intermediate in the phosphatidylcholine resynthesis pathway and in the mitochondrial NADPH production pathway of the cofactor, carrier, and vitamin biosynthesis superfamily. In addition, the parishin treatment-improved metabolite β-sitosterol is a plant-derived natural bioactive compound with various pharmacological activities, such as hypolipidemic, anti-inflammatory, antitumor, tissue repair and antiaging activities (Haiyuan et al., 2019; Cheng et al., 2020), and is used to treat fatty liver, type II diabetes, and arthritis (Gumede et al., 2020; Ramalingam et al., 2020). The positive correlation between β-sitosterol and bacterial taxa from *Prevotellaceae* has been reported in different animal models (Xia et al., 2020; Yu et al., 2021), which is consistent with our findings. However, the mechanism by which the gut microbiota regulates β-sitosterol metabolism is still unclear and needs to be further studied. Besides, the results of correlation analysis also showed that 3-(3-hydroxyphenyl)-propanoic acid and 4-hydroxybenzeneacetic acid were negatively correlated with GDF15. Fecal metabolites act as a bridge for the interaction between the host and gut microbes. These results suggest that parishin treatment may improve aging-induced gut metabolism disorders in the host to exert health-promoting effects by remodeling the gut microbiota.

In conclusion, this study describes the health-promoting effects of parishin on aged mice. Parishin significantly decreased aging-induced GDF15, IL-6 and p16^*Ink*4*a*^ levels, improved dysbiosis in the gut microbiota and metabolome, reduced pathological abnormalities in cardiopulmonary tissues, and has important potential applications against aging.

## Data Availability Statement

The datasets presented in this study can be found in online repositories. The names of the repository/repositories and accession number(s) can be found in the article/supplementary material.

## Ethics Statement

The animal study was reviewed and approved by Animal Experimentation Ethics Committee of Zhejiang University.

## Author Contributions

SZ, XZ, and YY conceived and designed the study. SZ, XZ, RY, CG, QG, QZ, LX, LC, PW, and SL performed the experiments and analyzed the data. SZ, XZ, and RY wrote the manuscript. All authors contributed to the article and approved the submitted version.

## Conflict of Interest

The authors declare that the research was conducted in the absence of any commercial or financial relationships that could be construed as a potential conflict of interest.

## Publisher’s Note

All claims expressed in this article are solely those of the authors and do not necessarily represent those of their affiliated organizations, or those of the publisher, the editors and the reviewers. Any product that may be evaluated in this article, or claim that may be made by its manufacturer, is not guaranteed or endorsed by the publisher.
